# Regularized EM algorithm for sparse parameter estimation in nonlinear dynamic systems with application to gene regulatory network inference

**DOI:** 10.1186/1687-4153-2014-5

**Published:** 2014-04-03

**Authors:** Bin Jia, Xiaodong Wang

**Affiliations:** 1 Intelligent Fusion Technology, Germantown, Inc., MD 20876, USA; 2 Department of Electrical Engineering, Columbia University, New York, NY 10027, USA

**Keywords:** Nonlinear dynamic system, Parameter estimation, Sparsity, Expectation-maximization, Forward-backward recursion, Gaussian approximation, Gene regulatory network

## Abstract

Parameter estimation in dynamic systems finds applications in various disciplines, including system biology. The well-known expectation-maximization (EM) algorithm is a popular method and has been widely used to solve system identification and parameter estimation problems. However, the conventional EM algorithm cannot exploit the sparsity. On the other hand, in gene regulatory network inference problems, the parameters to be estimated often exhibit sparse structure. In this paper, a regularized expectation-maximization (rEM) algorithm for sparse parameter estimation in nonlinear dynamic systems is proposed that is based on the maximum *a posteriori* (MAP) estimation and can incorporate the sparse prior. The expectation step involves the forward Gaussian approximation filtering and the backward Gaussian approximation smoothing. The maximization step employs a re-weighted iterative thresholding method. The proposed algorithm is then applied to gene regulatory network inference. Results based on both synthetic and real data show the effectiveness of the proposed algorithm.

## 1 Introduction

The dynamic system is a widely used modeling tool that finds applications in many engineering disciplines. Techniques for state estimation in dynamic systems have been well established. Recently, the problem of *sparse* state estimate has received significant interest. For example, various approaches to *static* sparse state estimation have been developed in [1-4], where the problem is essentially an underdetermined inverse problem, i.e., the number of measurements is small compared to the number of states. Extensions of these methods for *dynamic* sparse state estimation have been addressed in [5-7].

The expectation-maximization (EM) algorithm has also been applied to solve the sparse state estimate problem in dynamic systems [8-12]. In particular, in [8-10], the EM algorithm is employed to update the parameters of the Bernoulli-Gaussian prior and the measurement noise. These parameters are then used in the generalized approximate message passing algorithm [8-10]. In [12,13], the EM algorithm is used to iteratively estimate the parameters that describe the prior distribution and noise variances. Moreover, in [14], the EM algorithm is used for blind identification, where the sparse state is explored. Note that in the above works, only *linear* dynamic systems are considered.

In this paper, we focus on the *sparse parameter* estimation problem instead of the sparse state estimation problem. We consider a general *nonlinear* dynamic system, where both the state equation and the measurement equation are parameterized by some unknown parameters which are assumed to be sparse. One particular application is the inference of gene regulatory networks. The gene regulatory network can be modeled by the state-space model [15], in which the gene regulations are represented by the unknown parameters. The gene regulatory network is known to be sparse due to the fact that a gene directly regulates or is regularized by a small number of genes [16-19]. The EM algorithm has been applied to parameter estimation in dynamic systems [20]. However, the EM algorithm cannot exploit the sparsity of the parameters. Here, we propose a regularized expectation-maximization (rEM) algorithm for sparse parameter estimation in nonlinear dynamic systems. Specifically, the sparsity of the parameters is imposed by a Laplace prior and we consider the approximate maximum *a posteriori* (MAP) estimate of the parameters. It should be emphasized that the proposed method is an approximate MAP-EM algorithm based on various Gaussian assumptions and quadrature procedures for approximating Gaussian integrals. Note that the MAP-EM algorithm may get stuck at local minima or saddle points. Similar to the conventional EM algorithm, the rEM algorithm also consists of an expectation step and a maximization step. The expectation step involves the forward Gaussian approximation filtering and the backward Gaussian approximation smoothing. The maximization step involves solving an *ℓ*_1_ minimization problem for which a re-weighted iterative thresholding algorithm is employed. To illustrate the proposed sparse parameter estimation method in dynamic systems, we consider the gene-regulatory network inference based on gene expression data.

The unscented Kalman filter has been used in the inference of gene regulatory network [15,21,22]. However, the methods proposed in [15,21,22] are fundamentally different with the method proposed in this paper. Firstly, the unscented Kalman filter is only used once in [15,21,22], while it is used in each iteration of the rEM algorithm in this paper. Secondly, not only the unscented Kalman filter but also the unscented Kalman smoother is used in our proposed rEM algorithm. In essence, only the observation before time *k* is used to the estimation at time *k* in the unscented Kalman filter. However, in our rEM algorithm, all observation data is used to the estimation at time *k* (by the unscented Kalman smoother). The fundamental difference between the proposed work and that of [9] is that the proposed work is for the sparse parameter estimation problem of the dynamic system, while that of [9] is only for the sparse parameter estimation of the static problem. In addition, a general nonlinear dynamic system is involved in our work and only linear system is involved in the work of [9]. The main difference between the proposed work and that of [23] is that the sparsity constraint is enforced. The main contribution of this paper is to use the sparsity-enforced EM algorithm to solve the sparse parameter estimation problem. In addition, the reweighted iterative threshold algorithm is proposed to solve the *ℓ*_1_ optimization algorithm. To the best knowledge of the authors, the proposed rEM with the reweighted iterative threshold optimization algorithm is innovative. Furthermore, we have systematically investigated the performance of the proposed algorithm and compared the results with other conventional algorithms.

The remainder of this paper is organized as follows. In Section 2, the problem of the sparse parameter estimation in dynamic systems is introduced and the regularized EM algorithm is formulated. In Section 3, the E-step of rEM that involves forward-backward recursions and Gaussian approximations is discussed. Section 4 discusses the *ℓ*_1_ optimization problem involved in the maximization step. Application of the proposed rEM algorithm to gene regulatory network inference is discussed in Section 5. Concluding remarks are given in Section 6.

## 2 Problem statement and the MAP-EM algorithm

We consider a general discrete-time nonlinear dynamic system with unknown parameters, given by the following state and measurement equations:

(1) $$\begin{array}{ll} x_{k} & =f(x_{k-1},\theta )+u_{k},\; \end{array}$$

(2) $$\begin{array}{ll} \text{and}\;\;y_{k} & =h(x_{k},\theta )+v_{k},\; \end{array}$$

where ***x***_*k*_ and ***y***_*k*_ are the state vector and the observation vector at time *k*, respectively; ***θ*** is the unknown parameter vector; ***f*** (·) and ***h*** (·) are two nonlinear functions; $u_{k}∼N(0,U_{k})$ is the process noise, and $v_{k}∼N(0,R_{k})$ is the measurement noise. It is assumed that both {***u***_*k*_} and {***v***_*k*_} are independent noise processes and they are mutually independent. Note that the nonlinear functions ***f*** and ***h*** are assumed to be differentiable.

Define the notation $Y^{k}≜[y_{1},\cdots \;,y_{k}]$. The problem considered in this paper is to estimate the unknown system parameter vector ***θ*** from the length-*K* measurement data ***Y***^*K*^. We assume that ***θ*** is *sparse*. In particular, it has a Laplacian prior distribution which is commonly used as a sparse prior,

(3) $$p(\theta )=\overset{m}{\underset{i=1}{\prod }}\frac{\lambda_{i}}{2}e^{-\lambda_{i}|\theta_{i}|}.$$

In the EM algorithm and the MAP-EM algorithm [23], given an estimate ***θ***^′^, a new estimate ***θ***^′′^ is given by

(4) $$\theta^{\prime \prime }=\underset{\theta }{\arg\;\max}\;Q(\theta ,\theta^{\prime }),$$

and

(5) $$\theta^{\prime \prime }=\underset{\theta }{\arg\;\max}\;\left[Q(\theta ,\theta^{\prime })+\log p(\theta )\right],$$

respectively.

Note that the regularized EM can be viewed as a special MAP-EM. To differentiate the sparsity-enforced EM algorithm from the general MAP-EM algorithm, rEM is used. In this paper, the following assumptions are made. (1) The probability density function of the state is assumed to be Gaussian. The Bayesian filter is optimal; however, exact finite-dimensional solutions do not exist. Hence, numerical approximation has to be made. The Gaussian approximation is frequently assumed due to the relatively low complexity and high accuracy [24-26]. (2) The integrals are approximated by various quadrature methods. Many numerical rules, such as Gauss-Hermite quadrature [25], unscented transformation [27], cubature rule [24], and the sparse grid quadrature [26], as well as the Monte Carlo method [28], can be used to approximate the integral. However, the quadrature rule is the best when computational complexity and accuracy are both considered [29].

We next consider the expression of the *Q*-function in (5). Due to the Markovian structure of the state-space model (1) to (2), we have

(6) $$p(X^{K},Y^{K}|\theta )=p(x_{1}|\theta )\overset{K}{\underset{k=2}{\prod }}p(x_{k}|x_{k-1},\theta )\overset{K}{\underset{k=1}{\prod }}p(y_{k}|x_{k},\theta ).$$

Therefore,

(7) $$\begin{array}{ll} Q(\theta ,\theta^{\prime }) & =\int \log p(X^{K},Y^{K}|\theta )p(X^{K}|Y^{K},\theta^{\prime })dX^{K}\\ & =\int \log p(x_{1}|\theta )p(x_{1}|Y^{K},\theta^{\prime })dx_{1}\\ & +\overset{K}{\underset{k=2}{\sum }}\int \underset{-\frac{1}{2}{\left(x_{k}-f(x_{k-1},\theta )\right)}^{T}\overset{-1}{\underset{k}{U}}(x_{k}-f(x_{k-1},\theta ))-c_{k}}{\underset{︸}{\log p(x_{k}|x_{k-1},\theta )}}p(x_{k},x_{k-1}|Y^{K},\theta^{\prime })dx_{k-1}dx_{k}\\ & +\overset{K}{\underset{k=1}{\sum }}\int \underset{-\frac{1}{2}{\left(y_{k}-h(x_{k},\theta )\right)}^{T}\overset{-1}{\underset{k}{R}}(y_{k}-h(x_{k},\theta ))-d_{k}}{\underset{︸}{\log p(y_{k}|x_{k},\theta )}}p(x_{k}|Y^{K},\theta^{\prime })dx_{k}, \end{array}$$

where $c_{k}≜\frac{1}{2}[\;\log|U_{k}|+\text{dim}(x_{k})\log(2\pi )]$ and $d_{k}≜\frac{1}{2}[\;\log|R_{k}|+\text{dim}(y_{k})\log(2\pi )]$. We assume that the initial state ***x***_1_ is independent of the parameter ***θ***. Hence, with the prior given in (3), the optimization in (5) can be rewritten as

(8) $$\begin{array}{ll} \theta^{\prime \prime } & =\underset{\theta }{\arg\;\max}\;\left[Q(\theta ,\theta^{\prime })+\log p(\theta )\right]\\ & =\underset{\theta }{\arg\;\min}\;\overset{K}{\underset{k=2}{\sum }}\left\{2c_{k}+\int \left[\left(x_{k}-f{\left(x_{k-1},\theta \right)}^{T}U_{k}^{-1}(x_{k}-f(x_{k-1},\theta \right)\right]\right)\\ & \;\times p\left((x_{k},x_{k-1}|Y^{K},\theta^{\prime })dx_{k-1}dx_{k}\right\}\\ & +\overset{K}{\underset{k=1}{\sum }}\left\{2d_{k}+\int \left[\left(y_{k}-h{\left(x_{k},\theta \right)}^{T}R_{k}^{-1}(y_{k}-h(x_{k},\theta \right)\right]p(x_{k}|Y^{K},\theta^{\prime })dx_{k}\right\}\\ & +2∥\lambda \circ \theta {∥}_{1}, \end{array}$$

where ***λ*** =[*λ*_1_,*λ*_2_,⋯,*λ*_*m*_]^*T*^, and ‘ ∘’ denotes the point-wise multiplication.

Note that in many applications, the unknown parameters ***θ*** are only related to the state equation, but not to the measurement equation. Therefore, the second term in (8) can be removed. In the next section, we discuss the procedures for computing the densities *p*(***x***_*k*_, ***x***_*k*−1_| ***Y***^*K*^, ***θ***^′^) and *p*(***x***_*k*_| ***Y***^*K*^, ***θ***^′^), the integrals, and the minimization in (8).

## 3 The E-step: computing the *Q*-function

We first discuss the calculation of the probability density functions of the states *p*(***x***_*k*_, ***x***_*k*−1_| ***Y***^*K*^, ***θ***^′^) and *p*(***x***_*k*_| ***Y***^*K*^, ***θ***^′^) in (8), which involves a forward recursion of a point-based Gaussian approximation filter to compute *p*(***x***_*k*_| ***Y***^*k*^, ***θ***^′^) and *p*(***x***_*k*+1_| ***Y***^*k*^, ***θ***^′^), *k*=1,2,...,*K*, and a backward recursion of a point-based Gaussian approximation smoother to compute *p*(***x***_*k*_, ***x***_*k*−1_| ***Y***^*K*^, ***θ***^′^) and *p*(***x***_*k*_| ***Y***^*K*^, ***θ***^′^), *k*=*K*,*K*−1,...,1. For notational simplicity, in the remainder of this section, we drop the parameter ***θ***^′^.

### 3.1 Forward recursion

The forward recursion is composed of two steps: prediction and filtering. Specifically, given the prior probability density function (PDF) *p*(***x***_*k*−1_| ***Y***^*k*−1^) at time *k*−1, we need to compute the predicted conditional PDF *p*(***x***_*k*_| ***Y***^*k*−1^); then, given the measurement ***y***_*k*_ at time *k*, we update the filtered PDF *p*(***x***_*k*_| ***Y***^*k*^). These PDF recursions are in general computationally intractable unless the system is linear and Gaussian. The Gaussian approximation filters are based on the following two assumptions: (1) Given ***Y***^*k*−1^, ***x***_*k*−1_ has a Gaussian distribution, i.e., $x_{k-1}|Y^{k-1}∼N({\hat{x}}_{k-1|k-1},P_{k-1|k-1})$; and (2) (***x***_*k*_, ***y***_*k*_) are jointly Gaussian, given ***Y***^*k*−1^.

It then follows that the predictive PDF is Gaussian, i.e., $x_{k}|Y^{k-1}∼N({\hat{x}}_{k|k-1},P_{k|k-1})$, with [24,26,27]

(9) $$\begin{array}{ll} {\hat{x}}_{k|k-1} & ≜E\{x_{k}|Y^{k-1}\}=E_{x_{k-1}|Y^{k-1}}\left\{f(x_{k-1})\right\},\; \end{array}$$

(10) $$\begin{array}{lll} P_{k|k-1} & ≜\text{Cov}\{x_{k}|Y^{k-1}\}=E_{x_{k-1}|Y^{k-1}}\; & \;\\ & \;\left\{\;(f(x_{k-1})-{\hat{x}}_{k|k-1})(f{\left(x_{k-1}-{\hat{x}}_{k|k-1}\right)}^{T}\;\right\}+U_{k-1},\; \end{array}$$

where $E_{x_{k-1}|Y^{k-1}}\left\{g(x_{k-1})\right\}=\int g(x)\phi (x;\;{\hat{x}}_{k-1|k-1},$ ***P***_*k*−1|*k*−1_)d ***x***, and $\phi \left(x;\;\hat{x},P\right)$ denotes the multivariate Gaussian PDF with mean $\hat{x}$ and covariance ***P***.

Moreover, the filtered PDF is also Gaussian, i.e., $x_{k}|Y^{k}∼N({\hat{x}}_{k|k},P_{k|k})$[24,26,27], where

(11) $$\begin{array}{ll} {\hat{x}}_{k|k} & ≜E\{x_{k}|Y^{k}\}={\hat{x}}_{k|k-1}+L_{k}(y_{k}-{\hat{y}}_{k|k-1}),\; \end{array}$$

(12) $$\begin{array}{ll} \text{and}\;\;\;P_{k|k} & ≜\text{Cov}\{x_{k}|Y^{k}\}=P_{k|k-1}-L_{k}P_{k}^{\text{xy}},\; \end{array}$$

with

(13) $$\begin{array}{ll} {\hat{y}}_{k|k-1} & =E_{x_{k}|Y^{k-1}}\left\{h(x_{k})\right\},\; \end{array}$$

(14) $$\begin{array}{ll} L_{k} & =P_{k}^{\text{xy}}{\left(R_{k}+P_{k}^{\text{yy}}\right)}^{-1},\; \end{array}$$

(15) $$\begin{array}{ll} P_{k}^{\text{xy}} & =E_{x_{k}|Y^{k-1}}\left\{(x_{k}-{\hat{x}}_{k|k-1}){\left(h(x_{k})-{\hat{y}}_{k|k-1}\right)}^{T}\right\},\; \end{array}$$

(16) $$\begin{array}{ll} P_{k}^{\text{yy}} & =E_{x_{k}|Y^{k-1}}\left\{(h(x_{k})-{\hat{y}}_{k|k-1}){\left(h(x_{k})-{\hat{y}}_{k|k-1}\right)}^{T}\right\}.\; \end{array}$$

### 3.2 Backward recursion

In the backward recursion, we compute the smoothed PDFs *p*(***x***_*k*_, ***x***_*k*+1_| ***Y***^*K*^) and *p*(***x***_*k*_| ***Y***^*K*^). Here, the approximate assumption made is that conditioned on ***y***^*k*^, ***x***_*k*_ and ***x***_*k*+1_ are jointly Gaussian [30], i.e.,

(17) $$\begin{array}{lll} & \left[\begin{array}{c} x_{k}\\ x_{k+1} \end{array}\right]|Y^{k}∼N\left(\left[\begin{array}{c} {\hat{x}}_{k|k}\\ {\hat{x}}_{k+1|k} \end{array}\right],\right)\; & \;\\ & \;\;\;\;\times \left(\left[\begin{array}{cc} P_{k|k} & C_{k}\\ C_{k}^{T} & P_{k+1|k} \end{array}\right]\right),\; \end{array}$$

(18) $$\begin{array}{lll} \text{with}\;C_{k} & ≜\text{Cov}\{x_{k},x_{k+1}|Y^{k}\}\; & \;\\ & =E_{x_{k}|Y_{k}}\left\{(x_{k}-{\hat{x}}_{k|k}){\left(f(x_{k})-{\hat{x}}_{k+1|k}\right)}^{T}\right\}.\; \end{array}$$

Due to the Markov property of the state-space model, we have *p*(***x***_*k*_| ***x***_*k*+1_, ***Y***^*K*^)=*p*(***x***_*k*_| ***x***_*k*+1_, ***Y***^*k*^). Therefore, we can write [30]

(19) $$\begin{array}{ll} p(x_{k},x_{k+1}|Y^{K}) & =p(x_{k}|x_{k+1},Y^{K})p(x_{k+1}|Y^{K})\\ & =p(x_{k}|x_{k+1},Y^{k})p(x_{k+1}|Y^{K}). \end{array}$$

Now, assume that

(20) $$x_{k+1}|Y^{K}∼N({\tilde{x}}_{k+1},{\tilde{P}}_{k+1}),\;\;\text{with}\;\;{\tilde{x}}_{K}={\hat{x}}_{K|K},\;\;{\tilde{P}}_{K}=P_{K|K}.$$

It then follows from (17) and (19) that [30]

(21) $$\left[\begin{array}{c} x_{k}\\ x_{k+1} \end{array}\right]|Y^{K}∼N\left(\left[\begin{array}{c} {\tilde{x}}_{k}\\ {\tilde{x}}_{k+1} \end{array}\right],\left[\begin{array}{cc} {\tilde{P}}_{k} & D_{k}{\tilde{P}}_{k+1}\\ {\tilde{P}}_{k+1}D_{k}^{T} & {\tilde{P}}_{k+1} \end{array}\right]\right),$$

where

(22) $$\begin{array}{ll} {\tilde{x}}_{k} & ={\hat{x}}_{k|k}+D_{k}({\tilde{x}}_{k+1}-{\hat{x}}_{k+1|k}),\; \end{array}$$

(23) $$\begin{array}{ll} {\tilde{P}}_{k} & =P_{k|k}+D_{k}({\tilde{P}}_{k+1}-P_{k+1|k})D_{k}^{T},\; \end{array}$$

(24) $$\begin{array}{ll} D_{k} & =C_{k}P_{k+1|k}^{-1}.\; \end{array}$$

### 3.3 Approximating the integrals

The integrals associated with the expectations in the forward-backward recursions for computing the approximate state PDFs, i.e., (9), (10), (13), (15), (16), and (18), as well as the integrals involved in computing the function *Q*(***θ***, ***θ***^′^) in (8), are integrals of Gaussian type that can be efficiently approximated by various quadrature methods. Specifically, if a set of weighted points {(***γ***_*i*_,*w*_*i*_),*i*=1,…,*N*} can be used to approximate the integral

(25) $$E_{N(0,I)}\{g(x)\}=\int g(x)\phi \left(x;0,I\right)dx\approx \overset{N}{\underset{i=1}{\sum }}w_{i}g(\gamma_{i}),$$

then the general Gaussian-type integral can be approximated by

(26) $$E_{N(\hat{x},P)}\{g(x)\}=\int g(x)\phi \left(x;\hat{x},P\right)dx\approx \overset{N}{\underset{i=1}{\sum }}w_{i}g(S\gamma_{i}+\hat{x}),$$

where ***P*** = ***S*** ***S***^*T*^ and ***S*** can be obtained by Cholesky decomposition or singular value decomposition (SVD).

By using different point sets, different Gaussian approximation filters and smoothers can be obtained, such as the Gauss-Hermite quadrature (GHQ) [25], the unscented transform (UT) [27], the spherical-radial cubature rule (CR) [24], the sparse grid quadrature rule (SGQ) [26], and the quasi Monte Carlo method (QMC) [28]. Both the UT and the CR are the third-degree numerical rules which means the integration can be exactly calculated when ***g*** (***x***) is a polynomial with the degree up to three. In addition, the form of the CR is identical to the UT with a specific parameter. The main advantage of the UT and the CR is that the number of points required by the rule increases linearly with the dimension. However, one problem of the UT and the CR is that the high-order information of the nonlinear function is difficult to capture so that the accuracy may be low when ***g*** (***x***) is a highly nonlinear function. The GHQ rule, in contrast, can capture arbitrary degree information of ***g*** (***x***) by using more points. It has been proven that GHQ can provide more accurate results than the UT or the CR [25,26]. Similarly, the QMC method can also obtain more accurate results than the UT. However, both the GHQ rule and the QMC method require a large number of points for the high-dimensional problem. Specifically, the number of points required by the GHQ rule increases exponentially with the dimension. To achieve a similar performance of the GHQ with a small number of points, the SGQ is proposed [26], where the number of points increases only polynomially with the dimension.

For the numerical results in this paper, the UT is used in the Gaussian approximation filter and smoother, where we have *N*=2*n*+1, with *n* being the dimension of the state vector ***x***_*k*_. The quadrature points and the corresponding weights are given, respectively, by

(27) $$\gamma_{i}=\left\{\begin{array}{lr} 0,\; & i=1,\\ \sqrt{\left(n+\kappa \right)}e_{i-1},\; & i=2,\cdots \;,n+1,\\ -\sqrt{\left(n+\kappa \right)}e_{i-n-1},\; & i=n+2,\cdots \;,2n+1, \end{array}\right)$$

and

(28) $$w_{i}=\left\{\begin{array}{lr} \frac{\kappa }{n+\kappa },\; & i=1,\\ \frac{1}{2(n+\kappa )},\; & i=2,\cdots \;,2n+1, \end{array}\right)$$

where *κ* is a tunable parameter, and ***e***_*i*_ is the *i*th *n* dimensional unit vector. Note that *κ*=0 is used as the default value in this paper, as in the cubature Kalman filter [24]. In addition, *κ*=−3 can also be used as in the unscented Kalman filter [27].

## 4 The M-step: solving the ***ℓ***_***1***_ optimization problem

Solving the *ℓ*_1_ optimization problems in (8) is not trivial since |*θ*_*i*_| is nondifferentiable at *θ*_*i*_=0. The *ℓ*_1_ optimization is a useful tool to obtain sparse solutions. Methods for solving linear inverse problems with sparse constraints are reviewed in [1]. Some more recent developments include the projected scaled subgradient [31] method, the gradient support pursuit method [32], and the greedy sparse-simplex method [33]. In this paper, for the maximization step in the proposed rEM algorithm, due to the simplicity of implementation, we will employ a modified version of the iterative thresholding algorithm.

### 4.1 Iterative thresholding algorithm

Denote $\tilde{Q}(\theta ,\theta^{\prime })$ as the two summation terms in (8). We consider the optimization problem in (8)

(29) $$\underset{\theta }{\arg\;\min}\;J(\theta )=\tilde{Q}(\theta ,\theta^{\prime })+2∥\lambda \circ \theta {∥}_{1}.$$

The solution to (29) can be iteratively obtained by solving a sequence of optimization problems [34]. As in the Newton’s method, the Taylor series expansion of the $\tilde{Q}(\theta ,\theta^{\prime })$ around the solution ***θ***^*t*^ at the *t*th iteration is given by

(30) $$\tilde{Q}(\theta^{t}+\Delta \theta ,\theta^{\prime })≅\tilde{Q}(\theta^{t},\theta^{\prime })+\Delta \theta^{T}\nabla \tilde{Q}(\theta^{t},\theta^{\prime })+\frac{\alpha_{t}}{2}∥\Delta \theta {∥}_{2}^{2},$$

where $\nabla \tilde{Q}$ is the gradient of the negative *Q*-function and *α*_*t*_ is such that *α*_*t*_ ***I*** mimics the Hessian $\nabla^{2}\tilde{Q}$. Then, ***θ***^*t*+1^ is given by

(31) $$\theta^{t+1}\;=\;\underset{z}{\arg\;\min}\;{\left(z-\theta^{t}\right)}^{T}\nabla \tilde{Q}(\theta^{t},\theta^{\prime })+\frac{\alpha_{t}}{2}∥z-\theta^{t}{∥}_{2}^{2}+2∥\lambda \circ z{∥}_{1},$$

where ***z*** denotes the variable to be optimized in the objective function.

The equivalent form of (31) is given by

(32) $$\begin{array}{ll} \theta^{t+1} & =\underset{z}{\arg\;\min}\;\frac{1}{2}∥z-u^{t}{∥}_{2}^{2}+\frac{2}{\alpha_{t}}∥\lambda \circ z{∥}_{1},\; \end{array}$$

(33) $$\begin{array}{ll} \text{with}\;u^{t} & =\theta^{t}-\frac{1}{\alpha_{t}}\nabla \tilde{Q}(\theta^{t},\theta^{\prime }),\; \end{array}$$

(34) $$\begin{array}{ll} \alpha_{t} & \approx \frac{{\left(s^{t}\right)}^{T}r^{t}}{∥s^{t}{∥}^{2}},\; \end{array}$$

(35) $$\begin{array}{ll} s^{t} & =\theta^{t}-\theta^{t-1},\; \end{array}$$

(36) $$\begin{array}{ll} r^{t} & =\nabla \tilde{Q}(\theta^{t},\theta^{\prime })-\nabla \tilde{Q}(\theta^{t-1},\theta^{\prime }).\; \end{array}$$

Note that Equation 34 is derived as follows. Because we require that *α*_*t*_ ***I*** mimics the Hessian $\nabla^{2}\tilde{Q}$, i.e., *α*_*t*_ ***s***^*t*^≈ ***r***^*t*^, solving *α*_*t*_ in the least-squares sense, we have

(37) $$\alpha_{t}\approx \underset{\alpha }{\arg\;\min}\;∥\alpha s^{t}-r^{t}{∥}_{2}^{2}=\frac{{\left(s^{t}\right)}^{T}r^{t}}{{s^{t})}^{T}s^{t}}.$$

The solution to (32) is given by $\theta^{t+1}=\eta^{S}(u^{t},\frac{2\lambda }{\alpha_{t}})$, where

(38) $$\eta^{S}(u^{t},a)=\text{sign}(u^{t})\text{max}\left\{|u^{t}|-a,0\right\}$$

is the soft thresholding function with sign(***u***^*t*^) and max{| ***u***^*t*^|−***a***, ***0***} being component-wise operators.

Finally, the iterative procedure for solving (29) is given by

(39) $$\begin{array}{lll} \theta^{t+1} & =\text{sign}\left(\theta^{t}-\frac{1}{\alpha_{t}}\nabla \tilde{Q}(\theta^{t},\theta^{\prime })\right)\; & \;\\ & \;\;\text{max}\left\{\left|\theta^{t}-\frac{1}{\alpha_{t}}\nabla \tilde{Q}(\theta^{t},\theta^{\prime })\right|-\frac{2\lambda }{\alpha_{t}},0\right\}.\; \end{array}$$

And the iteration stops when the following condition is met:

(40) $$\frac{\left|J(\theta^{t+1})-J(\theta^{t})\right|}{\left|J(\theta^{t})\right|}\leq \varepsilon ,$$

where *ε* is a given small number.

### 4.2 Adaptive selection of ***λ***

So far, the parameters *λ*_*i*_ in the Laplace prior are fixed. Here, we propose to adaptively tune them based on the output of the iterative thresholding algorithm. The algorithm consists of solving a sequence of weighted *ℓ*_1_-minimization problems. *λ*_*i*_ used for the next iteration are computed from the value of the current solution. A good choice of *λ*_*i*_ is to make them counteract the influence of the magnitude of the *ℓ*_1_ penalty function [35]. Following this idea, we propose an iterative re-weighted thresholding algorithm. At the beginning of the maximization step, we set *λ*_*i*_=1,∀*i*. Then, we run the iterative thresholding algorithm to obtain ***θ***. Next, we update *λ*_*i*_ as $\lambda_{i}=\frac{1}{|\theta_{i}|+\varepsilon },\forall i$, where *ε* is a small positive number, and run the iterative thresholding algorithm again using the new ***λ***. The above process is repeated until it converges at the point where the maximization step completes. Note that for the iterative re-weighted thresholding algorithm, the assumption that *θ* has a Laplacian prior no longer holds.

## 5 Application to gene regulatory network inference

The gene regulatory network can be described by a graph in which genes are viewed as nodes and edges depict causal relations between genes. By analyzing collected gene expression levels over a period of time, one can find some regulatory relations between different genes. Under the discrete-time state-space modeling, for a gene regulatory network with *n* genes, the state vector ***x***_*k*_=[*x*_1,*k*_,…,*x*_*n*,*k*_]^*T*^, where *x*_*i*,*k*_, denotes the gene expression level of the *i*th gene at time *k*.

In this case, the nonlinear function ***f*** (***x***) in the general dynamic Equation (1) is given by [15]

(41) $$f(x_{k-1},\theta )=Ag(x_{k-1}),$$

with

(42) $$g(x)=\left[\begin{array}{c} g(x_{1})\\ \vdots \\ g(x_{n}) \end{array}\right],$$

and

(43) $$g_{i}(x)=\frac{1}{1+e^{-x}},\;\forall i=1,\cdots \;,\mathrm{n.}$$

In (41), ***A*** is an *n*×*n* regulatory coefficient matrix with the element *a*_*i* *j*_ denoting the regulation coefficient from gene *j* to gene *i*. A positive coefficient *a*_*i* *j*_ indicates that gene *j* activates gene *i*, and a negative *θ*_*i* *j*_ indicates that gene *j* represses gene *i*. The parameter to be estimated is ***θ*** = ***A*** which is sparse.

For the measurement model, we have

(44) $$y_{k}=x_{k}+v_{k}.$$

### 5.1 Inference of gene regulatory network with four genes

In the simulations, we consider a network with four genes. The true gene regulatory coefficients matrix is given by

(45) $$A=\left[\begin{array}{cccc} 3 & 0 & 0 & -4.5\\ -2.9 & 0 & 5 & 0\\ -6 & 4 & 0 & 0\\ 0 & -5 & 2 & 0 \end{array}\right].$$

To compare the EM algorithm with the proposed rEM algorithm, the simulation was conduced ten times. In each time, the initial value of ***A*** (***θ***) is randomly generated from a Gaussian distribution with mean 0 and variance 2. The EM, rEM, and rEM_*w*_, as well as the basis pursuit de-noising dynamic filtering (BPDN-DF) method and the *ℓ*_1_ optimization method, are tested. Here, rEM_*w*_ denotes the version of the rEM algorithm with the iterative re-weighted thresholding discussed in Section 4.2.

As a performance metrics, the receiver operating characteristic (ROC) curve is frequently used. However, for this specific example, with the increasing of the false-positive rate, the true-positive rate given by rEM and EM is always high (close to 1) which makes the distinguishment of the performance of rEM algorithm and EM algorithm difficult. Hence, the root mean-squared error (RMSE) and the sparsity factor (SF) are used in this section. The RMSE is defined by

(46) $$\text{RMSE}=\sqrt{\frac{1}{N^{2}}\overset{N}{\underset{i=1}{\sum }}\overset{N}{\underset{j=1}{\sum }}{\left(A_{\text{ij}}-{\bar{A}}_{\text{ij}}\right)}^{2}},$$

where $\bar{A}$ denotes the estimated ***A***. The SF is given by

(47) $$\text{SF}=\frac{\phi_{0}}{\phi },$$

where *ϕ*_0_ and *ϕ* are the number of zero values of the estimated parameter and the number of zero values of true parameter, respectively. It can be seen that the estimation is over sparse if the sparsity factor is greater than 1.

In addition, to test the effectiveness of the proposed method at finding the support of the unknown parameters, the number of matched elements is used and can be obtained by the following procedures: (1) Compute the support of ***A*** using the true parameters (denoted by ***A***_*s*_) and the support of ***A*** using the estimated parameters (denoted by ${\bar{A}}_{s}$). Note that we assign [ ***A***_*s*_]_*i* *j*_=1 if *A*_*i* *j*_≠0 and [ ***A***_*s*_]_*i* *j*_=0 if *A*_*i* *j*_=0. Similarly, we assign ${\left[\;{\bar{A}}_{s}\right]}_{\text{ij}}=1$ if ${Ā}_{\text{ij}}\neq 0$ and ${\left[\;{\bar{A}}_{s}\right]}_{\text{ij}}=0$ if ${Ā}_{\text{ij}}=0$. (2) Compute the number of zero elements of $A_{s}-{\bar{A}}_{s}$ as the matched elements. It is easy to see that it is effective at finding the support of the unknown parameters when the number of matched elements is large.

#### 5.1.1 The effect of different *λ*

**Figure 1:**
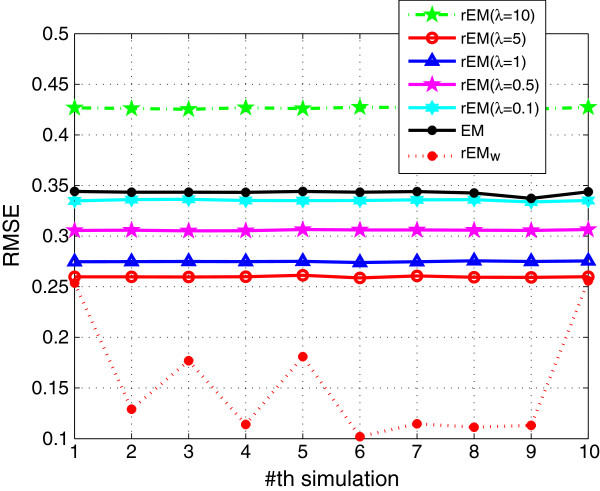
**RMSE of rEM with different *λ *** **and rEM**_***w***_**.**

**Figure 2:**
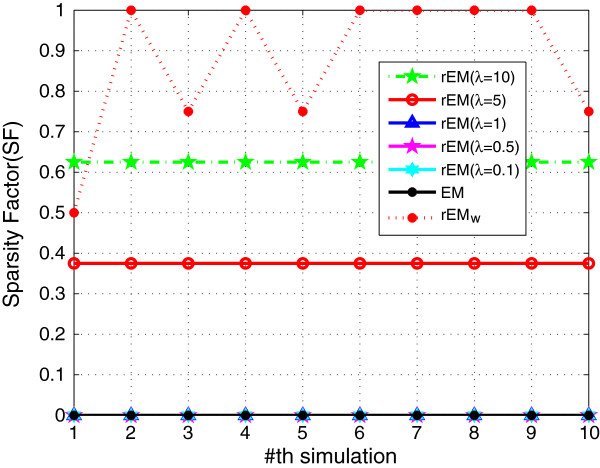
**SF of rEM with different *λ *** **and rEM**_***w***_**.**

**Figure 3:**
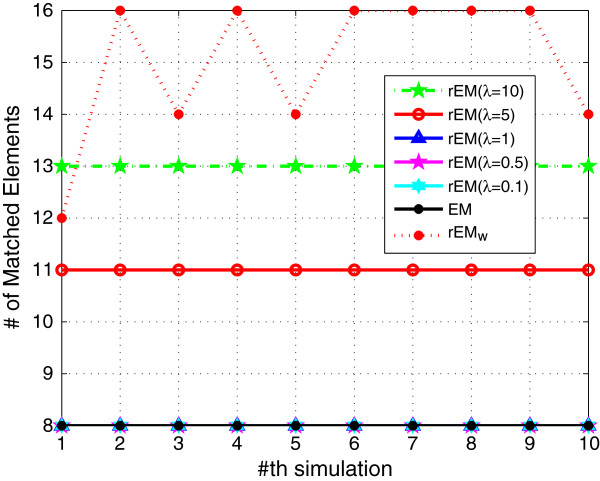
**The number of matched elements of rEM with different *λ *** **and rEM**_***w***_**.**

The performance of rEM using different *λ* (10,5,1, 0.5,0.1) is compared with the EM algorithm and the rEM_*w*_. The RMSE and SF are shown in Figures 1 and 2, respectively. The RMSE does not increase monotonously with the decreasing of parameter *λ*. It can be seen that the rEM with *λ*=5 has better performance than that using other *λ*. In addition, the rEM with all *λ* except *λ*=10 outperforms the EM algorithm. It provides smaller RMSE and sparser result. The rEM_*w*_ provides the smallest RMSE and sparsest parameter estimation. The number of matched elements of test algorithms with different *λ* is given in Figure 3. It can also be seen that rEM_*w*_ provides more matched elements than the EM algorithm.

#### 5.1.2 The effect of noise

**Figure 4:**
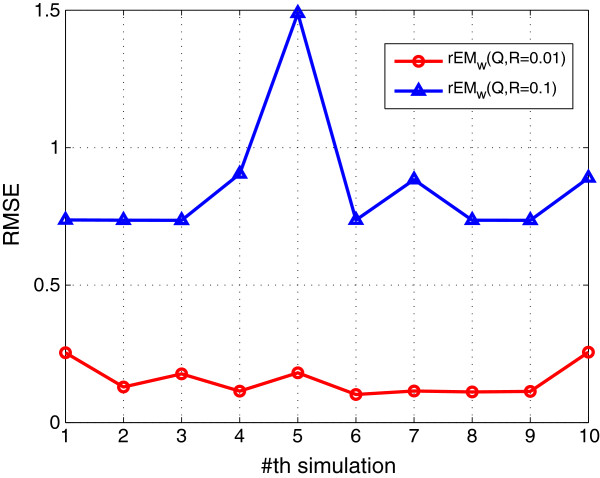
**RMSE of rEM**_***w ***_**with different noise levels.**

**Figure 5:**
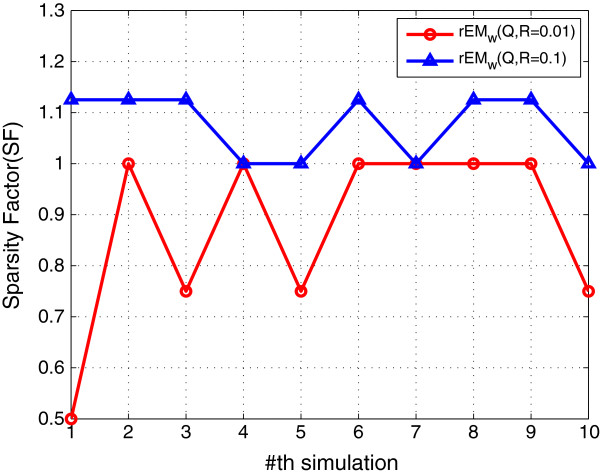
**SF of rEM**_***w ***_**with different noise levels.**

**Figure 6:**
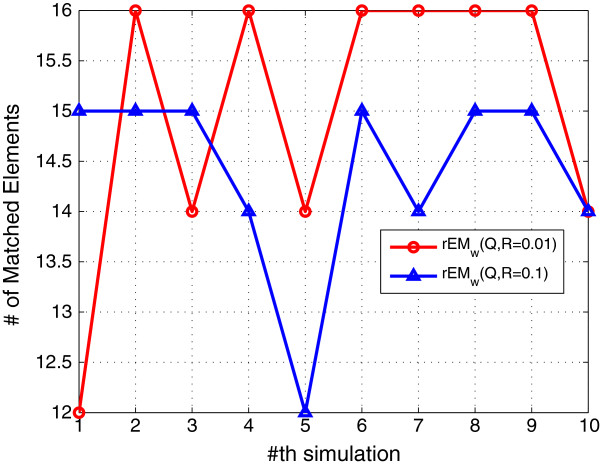
**The number of matched elements of rEM**_***w ***_**with different noise levels.**

Two different cases are tested. In the first case, the covariance of the process noise and measurement noise are chosen to be 0.01. In the second case, they are chosen to be 0.1. The performance of two test cases is shown in Figures 4, 5, 6. It can be seen that the RMSE of rEM_*w*_ with ***U***, ***R*** =0.01 ***I*** is smaller than that with ***U***, ***R*** =0.1 ***I***. In addition, the rEM_*w*_ with ***U***, ***R*** =0.01 provides a larger number of matched elements than that with ***U***, ***R*** =0.1 as shown in Figure 6. Hence, the estimation accuracy is better when the process noise and measure noise are small.

#### 5.1.3 The effect of the number of observations

**Figure 7:**
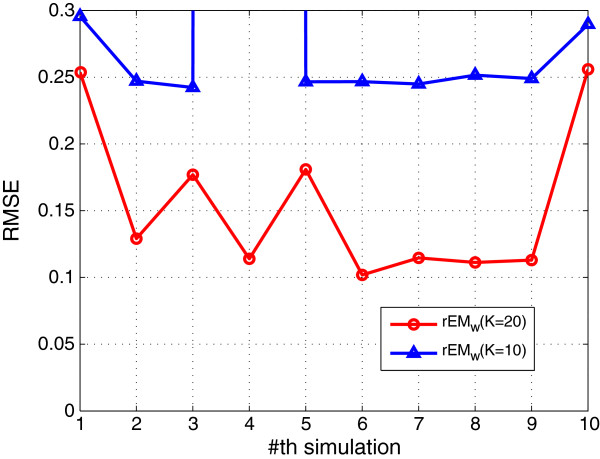
**RMSE of rEM**_***w ***_**with different lengths of observations.**

**Figure 8:**
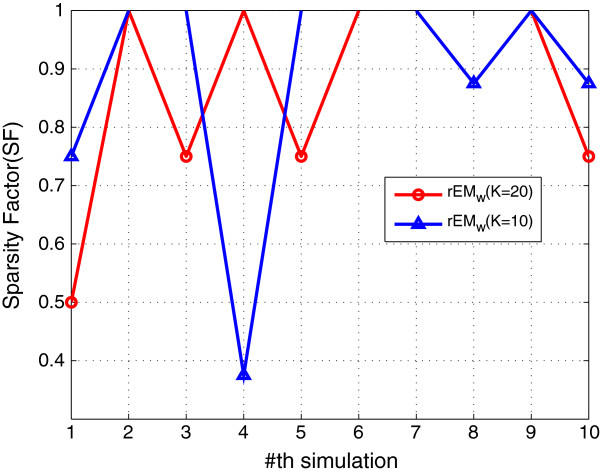
**SF of rEM**_***w ***_**with different lengths of observations.**

**Figure 9:**
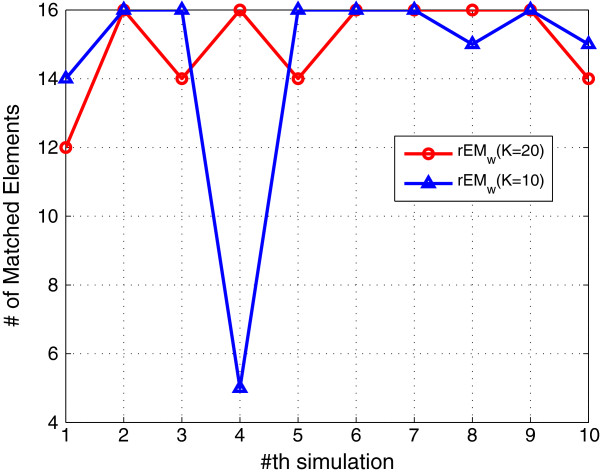
**The number of matched elements of rEM**_***w ***_**with different lengths of observations.**

In order to test the effect of the number of observations, the rEM_*w*_ algorithm with 10 and 20 observations are tested. The simulation results are shown in Figures 7, 8, 9. It can be seen that rEM_*w*_ with more observations gives less RMSE. In addition, as shown in Figure 9, rEM_*w*_ with more observations gives slightly better result for finding the support of the unknown parameters.

#### 5.1.4 The effect of ***κ***

**Figure 10:**
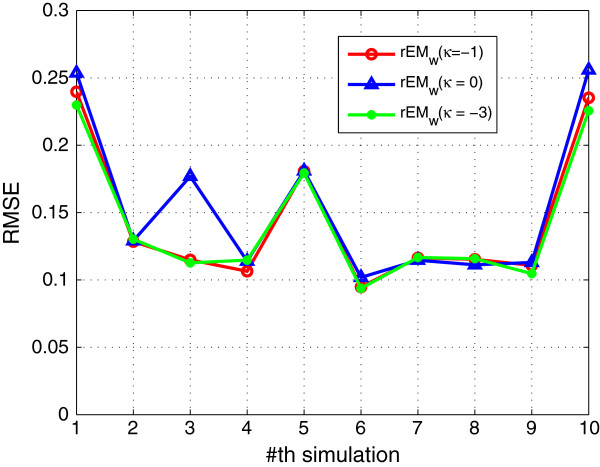
**RMSE of rEM**_***w ***_**with different *κ*.**

**Figure 11:**
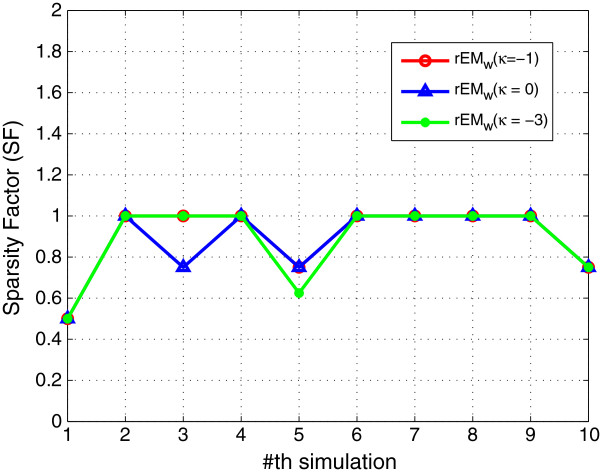
**SR of rEM**_***w ***_**with different *κ*.**

**Figure 12:**
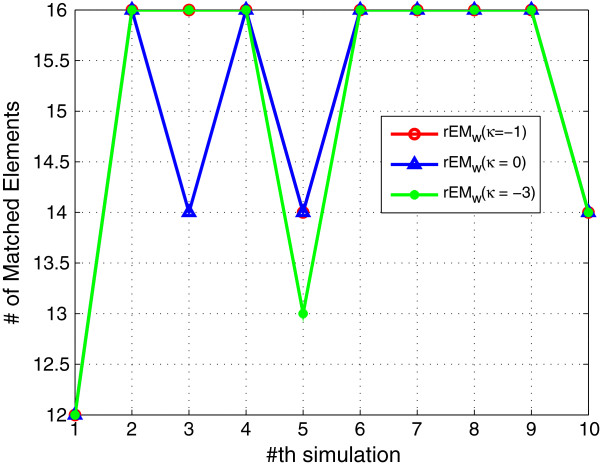
**The number of matched elements of rEM**_***w ***_**with different *κ*.**

In order to test the performance of *κ*, the rEM_*w*_ algorithm with different *κ* (0,-1,-3) is tested. The performance results are shown in Figures 10, 11, 12. Note that the cubature rule corresponds to *κ*=0, and the unscented transformation corresponds to *κ*=−1. Roughly speaking, the performance of rEM_*w*_ with different *κ* is close. Specifically, it can be seen that the RMSE of rEM_*w*_ using *κ*=−1 and rEM_*w*_ using *κ*=−3 is less than that of rEM_*w*_ using *κ*=0. The sparsity factor of rEM_*w*_ using *κ*=−1 is more close to 1 than that of rEM_*w*_ using *κ*=−3 and *κ*=0. Moreover, the number of matched elements of rEM_*w*_ using *κ*=−1 is more than that of rEM_*w*_ using *κ*=−3 and *κ*=0. Hence, the performance of rEM using *κ*=−1 is the best in this case.

#### 5.1.5 Effect of sparsity level

**Figure 13:**
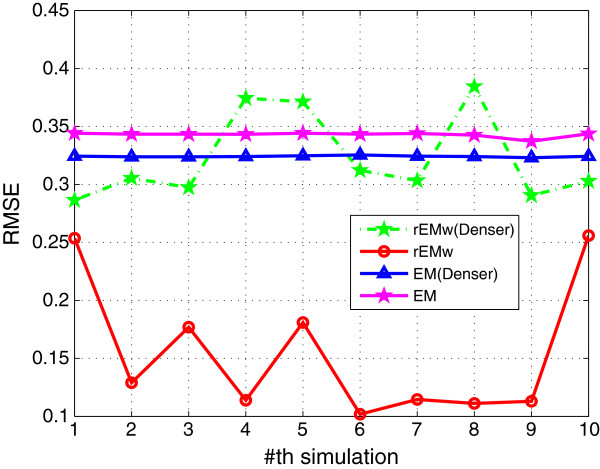
**RMSE of the EM and rEM**_***w ***_**for normal and denser *A*.**

**Figure 14:**
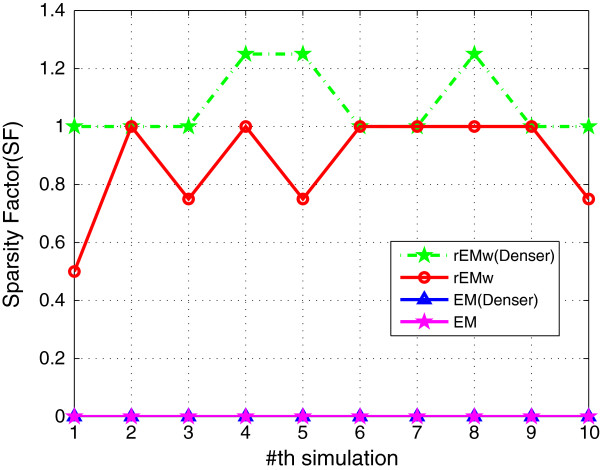
**SF of the EM and rEM**_***w ***_**for normal and denser *A*.**

**Figure 15:**
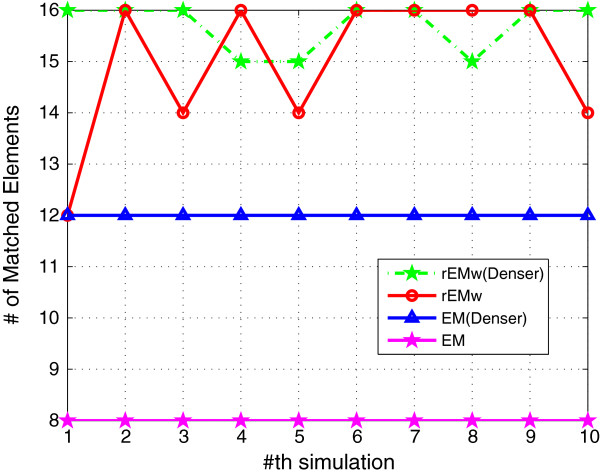
**The number of matched elements of the EM and rEM**_***w ***_**for normal and denser *A*.**

The performance comparison of the rEM_*w*_ and the conventional EM with different sparsity levels of ***A*** is shown in Figures 13, 14, 15. In this subsection, another ***A*** which is denser than the previously used ***A*** is given by

(48) $$A=\left[\begin{array}{cccc} 3 & -1 & 0 & -4.5\\ -2.9 & 0 & 5 & 1\\ -6 & 4 & 0 & -1\\ 1 & -5 & 2 & 0 \end{array}\right].$$

Note that ‘(Denser)’ is used to denote the result using ***A*** shown in Equation 48. It can be seen that the RMSE of rEM_*w*_(Denser) is comparable to that of the EM(Denser). However, the sparsity factor of rEM_*w*_(Denser) is closer to 1 than that of the EM(Denser) which means that the rEM_*w*_(Denser) is better. In addition, the number of matched elements of the rEM_*w*_(Denser) is large than that of the EM(Denser), which means that the rEM_*w*_(Denser) is better than the EM(Denser) in finding the support of the unknown parameters. The performance of the rEM_*w*_(Denser), however, is worse than that of the rEM_*w*_ in terms of the improvement of the RMSE. Hence, the rEM algorithm may have close performance with the EM algorithm when the sparsity is not obvious.

#### 5.1.6 Comparison with ***ℓ***_***1***_ optimization

We compare the proposed rEM algorithm and the *ℓ*_1_ optimization-based method, as well as the conventional EM algorithm. The *ℓ*_1_ optimization is a popular approach to obtain the sparse solution. For the problem under consideration, it obtains an estimate of ***θ*** by solving the following optimization problem:

(49) $$\hat{\theta }\;=\;\underset{\theta }{\arg\;\min}\;\overset{K}{\underset{k=2}{\sum }}{\left[y_{k}-A(\theta )g({\hat{x}}_{k-1})\right]}^{T}\;[y_{k}-A(\theta )g({\hat{x}}_{k-1})]+\lambda ∥\theta {∥}_{1},$$

where ${\hat{x}}_{1}=x_{1}$ and ${\hat{x}}_{k+1}=g({\hat{x}}_{k})$.

**Figure 16:**
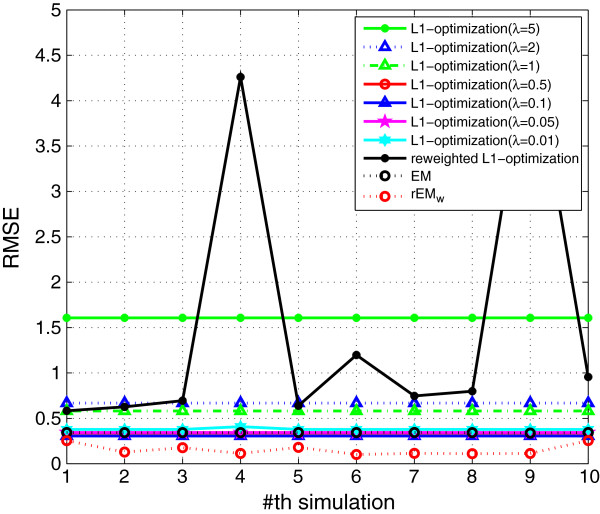
**RMSE of the *ℓ***_***1 ***_**optimization with different *λ*, reweighted *ℓ***_***1 ***_**optimization, EM, and rEM**_***w***_**.**

**Figure 17:**
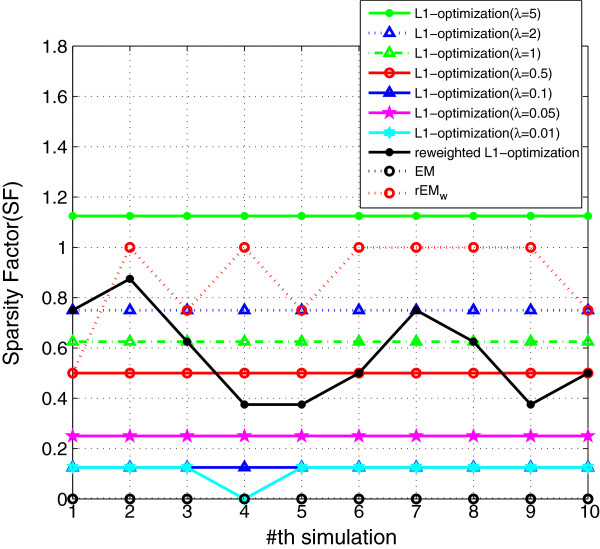
**SF of the *ℓ***_***1 ***_**optimization with different *λ*, reweighted *ℓ***_***1 ***_**optimization, EM, and rEM**_***w***_**.**

**Figure 18:**
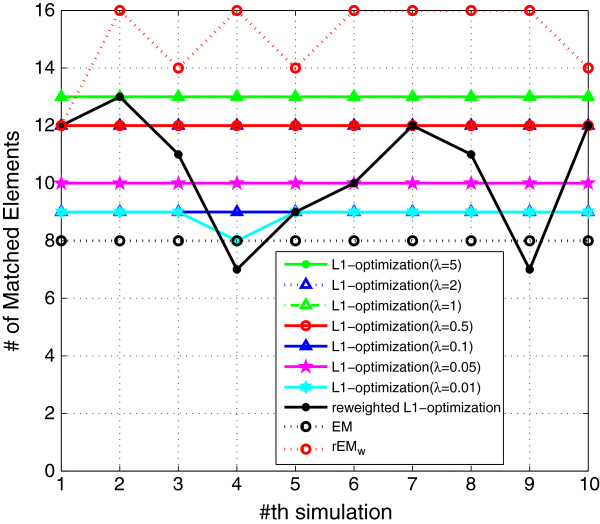
**The number of matched elements of the *ℓ***_***1 ***_**optimization with different *λ*, reweighted *ℓ***_***1 ***_**optimization, EM, and rEM**_***w***_**.**

We also compare the *ℓ*_1_ optimization method with the proposed rEM_w_ algorithm, and the results are shown in Figures 16, 17, 18. Seven different *λ* (5, 2, 1, 0.5, 0.1, 0.05, and 0.01) are used in the *ℓ*_1_ optimization method. The RMSE does not decrease monotonously with the decreasing of the parameter *λ*. Among all tested values, the *ℓ*_1_ optimization method with *λ*=0.1 gives the smallest RMSE. However, the sparsity factor of the *ℓ*_1_ optimization with *λ*=0.1 is far from the ideal value 1. The *ℓ*_1_ optimization with *λ*=5 gives the best support detection as shown in Figure 18. The re-weighted *ℓ*_1_ optimization algorithm is also used in the simulation. However, all *ℓ*_1_ optimization-based methods cannot achieve better performance than that of using the rEM_*w*_.

#### 5.1.7 Comparison with BPDN-DF

**Figure 19:**
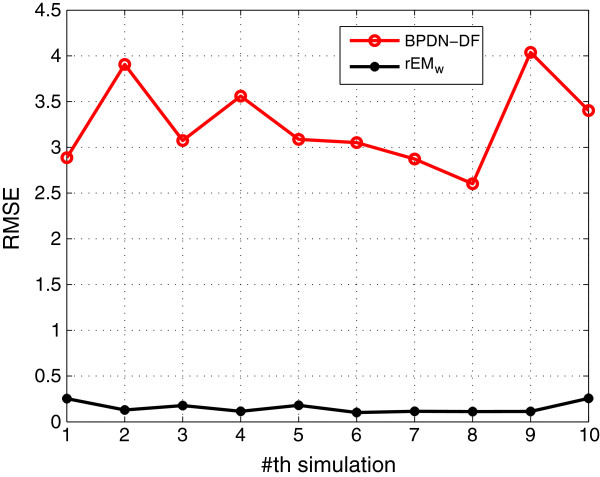
**RMSE of BPDN-DF and rEM**_***w***_**.**

**Figure 20:**
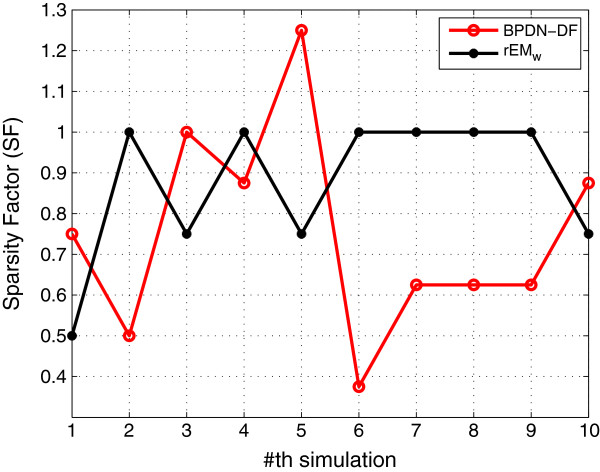
**SF of BPDN-DF and rEM**_***w***_**.**

**Figure 21:**
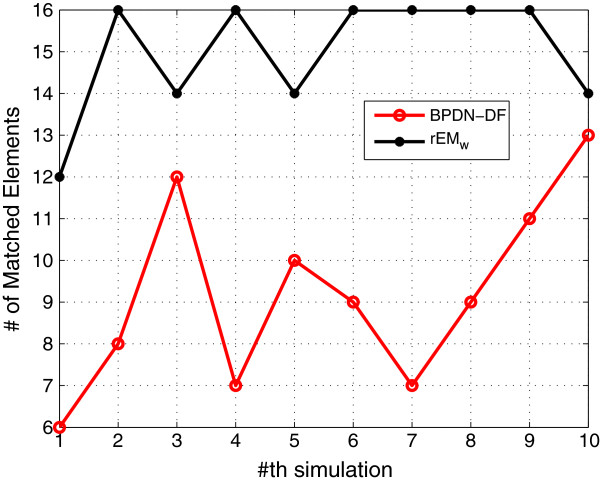
**The number of matched elements of BPDN-DF and rEM**_***w***_**.**

To solve the problem using BPDN-DF, the model in (41) and (44) are modified as

(50) $${\tilde{x}}_{k}=\tilde{f}({\tilde{x}}_{k-1})\left[\begin{array}{c} A(\theta_{k-1})g(x_{k-1})\\ \theta_{k-1} \end{array}\right]+\left[\begin{array}{c} v_{k}\\ 0 \end{array}\right]$$

and

(51) $$h({\tilde{x}}_{k})={\tilde{H}}_{k}+n_{k}=\left[\begin{array}{c} I_{4}0_{16} \end{array}\right]{\tilde{x}}_{k}+n_{k},$$

respectively. Note that ${\tilde{x}}_{k}={\left[x_{k}^{T},\theta_{k}^{T}\right]}^{T}$. Then, ${\hat{\tilde{x}}}_{k}$ is given by [36]

(52) $${\hat{\tilde{x}}}_{k}=\underset{\tilde{x}}{\arg\;\min}\;\left[∥y_{k}-{\tilde{H}}_{k}\tilde{x}{∥}_{2}^{2}+\lambda ∥\tilde{x}{∥}_{1}+∥\tilde{x}-\tilde{f}({\tilde{x}}_{k-1}){∥}_{2}^{2}\right],$$

where ***λ*** =[*λ*_1_,⋯,*λ*_20_] with *λ*_*i*_=0, *i*=1,2,3,4 since our objective is to explore the sparsity of the parameter ***θ***. The exact same initial values used in testing EM and rEM are used to test the performance of the BPDN-DF. The simulation results are shown in Figures 19, 20, 21. It can be seen that although the sparsity factor of BPDN-DF is comparable with that of the rEM_*w*_, the RMSE of the BPDN-DF is much larger than that of the rEM_w_. In addition, as shown in Figure 21, the rEM_w_ is better than the BPDN-DF in finding the support of the unknown parameters. The possible reason is that the BPDN-DF does not consider the noise in the dynamic system, and the measurement matrix ${\tilde{H}}_{k}$ is an ill-conditioned matrix. In the simulation, *λ*_*j*_=0.1, *j*=5,⋯,20. Based on our tests by using other values of *λ*, there is no obvious improvement.

### 5.2 Inference of gene regulatory network with eight genes

In this section, we test the proposed algorithm using a larger gene regulatory network which includes eight genes; the performances of the EM, the rEM, the rEM_*w*_, the *ℓ*_1_ optimization method, and BPDN-DF are given. Forty data points are collected to infer the structure of the network. The system noise and measurement noise are assumed to be Gaussian-distributed with means ***0*** and covariances ***U***_*k*_=0.01 ***I***_8_ and ***R***_*k*_=0.01 ***I***_8_, respectively. The connection coefficient matrix is given by

(53) $$A=\left(\begin{array}{cccccccc} 0 & 0 & 0 & 0 & 0 & 0 & 2.4 & 3.2\\ 0 & 0 & 0 & 4.1 & 0 & -2.4 & 0 & 4.1\\ -5.0 & 2.1 & -1.5 & 0 & 4.5 & 0 & 2.1 & 0\\ 0 & 1.3 & 2.5 & -3.7 & 1.8 & 0 & 0 & -3.1\\ 0 & 0 & 0 & -2.6 & -3.2 & 0 & -1 & 4\\ -1.5 & -1.8 & 0 & 3.4 & 1.4 & 1.1 & 0 & 1.7\\ -1.8 & 0 & 0 & -3 & 1.1 & 2.4 & 0 & 0\\ -1.3 & 0 & -1 & 0 & 2.1 & 0 & 0 & 2.2 \end{array}\right).$$

For testing, each coefficient in $\hat{A}$ is initialized from a Gaussian distribution with mean 0 and variance 1. The system state is initialized using the first measurement.

The metric used to evaluate the inferred GRN is the ROC curve, in which the true-positive rate (TPR) and the false-positive rate (FPR) are involved. They are given by

(54) $$\text{TPR}=\frac{\text{TP}\#}{\text{TP}\#+\text{FN}\#},$$

(55) $$\text{FPR}=\frac{\text{FP}\#}{\text{FP}\#+\text{TN}\#},$$

where the number of true positives (TP *#*) denotes the number of links correctly predicted by the inference algorithm, the number of false positives (FP *#*) denotes the number of incorrectly predicted links. The number of true negatives (TN *#*) denotes the number of correctly predicted non-links, and the number of false negatives (FN *#*) denotes the number of missed links by the inference algorithm [15].

**Figure 22:**
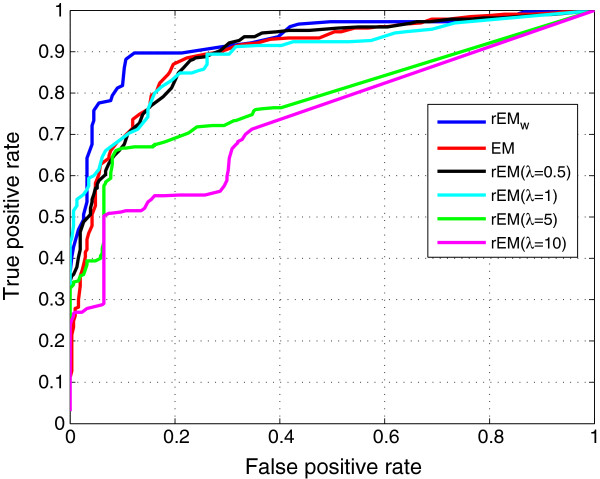
**ROCs of the EM, the rEM, and the rEM**_***w***_**.**

The ROC curves of the EM, the rEM, and the rEM_*w*_ are compared in Figure 22. The rEM with different *λ* is tested. In Figure 22, the curves of rEM with four typical values of *λ* are shown. There is no obvious improvement by using other *λ*. From the figure, it can be seen that the rEM_*w*_ performs better than the rEM and the convectional EM algorithms.

**Figure 23:**
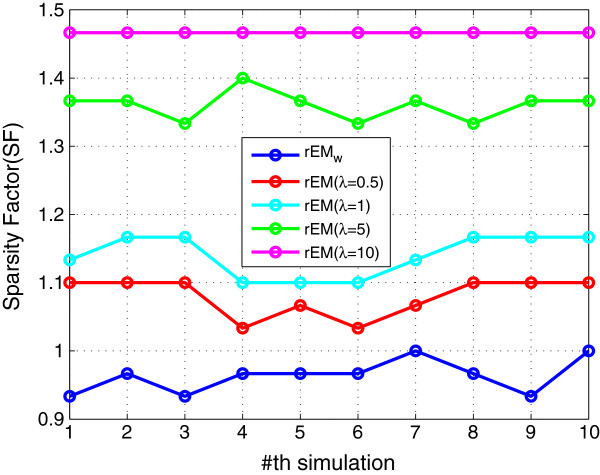
**Sparsity factor of the rEM and the rEM**_***w***_**.**

In addition, the sparse solution is obtained by using rEM and rEM_*w*_ while it cannot be obtained by using the EM algorithm. The sparsity factor of rEM and rEM_*w*_ is shown in Figure 23; the sparsity of the solution given by rEM_*w*_ is closer to the ground truth than that given by the EM algorithm.

**Figure 24:**
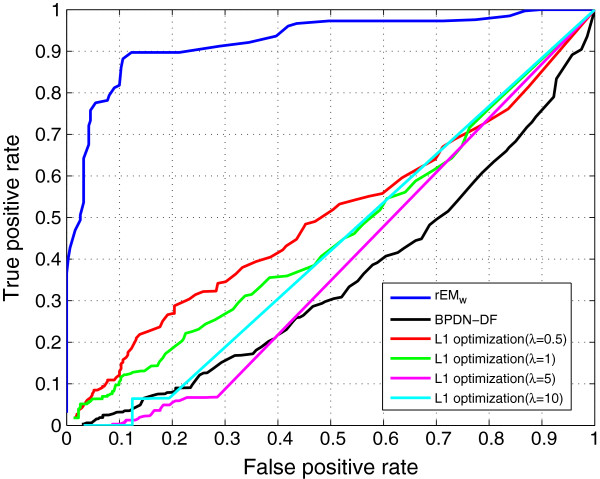
**ROCs of the rEM**_***w***_**, *ℓ***_***1 ***_**optimization method, and BPDN-DF.**

In Figure 24, the ROC curves of the rEM_*w*_, *ℓ*_1_ optimization method, and BPDN-DF are compared. Similarly, the *ℓ*_1_ optimization method with different *λ* is tested, and only four curves are shown in the figure. By using other values, there is no obvious improvement. The BPDN-DF with different *λ* has no obvious difference in the test. From Figure 24, it can be seen that the rEM_*w*_ performs much better than the *ℓ*_1_ optimization method and BPDN-DF algorithm. Hence, the sparsity factor of *ℓ*_1_ optimization method and BPDN-DF is not shown.

### 5.3 Inference of gene regulatory network from malaria expression data

The dataset with the first six gene expression data of malaria is given in reference [37] and is used in this section. The initial covariance for the algorithm is ***P***_0_=0.5 ***I***. The process noise and measurement noise are assumed to be Gaussian noise with zero mean and covariance 0.3^2^ ***I*** and 0.4^2^ ***I***, respectively. In the following, we show the inference results of the parameter and the state estimation provided by the unscented Kalman filter (UKF) based on the model using the inferred parameters.

The inferred ***A*** by the EM algorithm is

(56) $$\begin{array}{c} \bar{A}=\left[\begin{array}{cccccc} 2.2120 & -7.9443 & 2.3843 & 6.1800 & -3.5269 & 2.8300\\ -0.6585 & -0.5319 & 0.5987 & 4.0023 & -2.8684 & 1.1167\\ 1.9022 & -9.1935 & 3.0504 & 7.9274 & -5.0037 & 3.4825\\ 1.8157 & -8.8003 & 3.4441 & 9.4813 & -7.1284 & 3.4345\\ 1.8413 & -8.3515 & 2.2789 & 5.3726 & -1.6722 & 2.5999\\ 2.1053 & -3.3850 & 3.4007 & 10.2753 & -12.3170 & 2.1100 \end{array}\right]. \end{array}$$

The inferred ***A*** by the rEM with *λ*=1 is

(57) $$\begin{array}{c} \bar{A}=\left[\begin{array}{cccccc} 0.8448 & -6.3169 & 0.8943 & 3.9423 & 0 & 2.8387\\ 0 & -0.2422 & 0 & 0.5051 & 0 & 1.3407\\ 0.1424 & -7.1799 & 0.7461 & 5.2535 & 0.3607 & 2.9298\\ 0.0048 & -8.1010 & 0.7851 & 5.3300 & 0 & 4.2157\\ 0.4022 & -6.9358 & 2.0375 & 4.0332 & 0 & 2.7372\\ 0 & -5.6613 & 0 & 4.3934 & -2.9426 & 6.3350 \end{array}\right]. \end{array}$$

The inferred ***A*** by the rEM_*w*_ is

(58) $$\begin{array}{c} \bar{A}=\left[\begin{array}{cccccc} 0.3662 & -7.5033 & 0 & 9.6020 & 0 & 0\\ -2.0531 & -1.1905 & 0 & 5.1439 & -0.0011 & 0\\ 0 & -9.0526 & 0 & 11.6504 & 0 & 0\\ 0 & -9.3419 & 0 & 14.4056 & -3.5361 & 1.0739\\ 0.0034 & -8.5250 & 0 & 11.0732 & 0 & 0\\ 0 & -3.8773 & 0.0025 & 13.1848 & -8.3610 & 1.4877 \end{array}\right]. \end{array}$$

**Figure 25:**
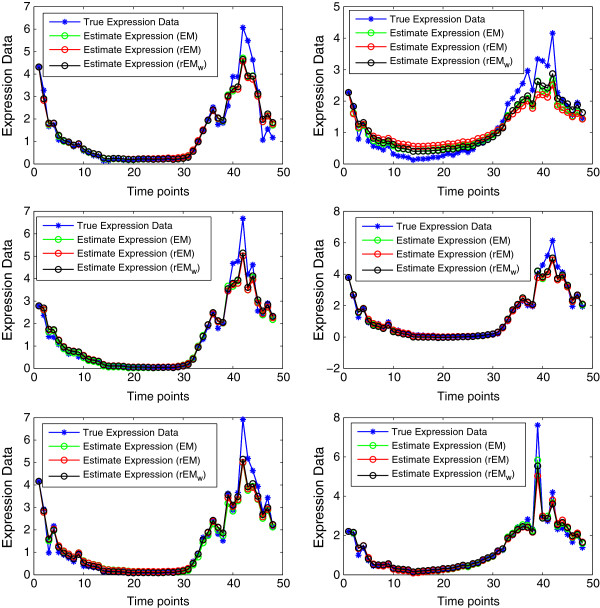
True malaria gene expression and estimated gene expression by different algorithms.

The state estimation provided by the UKF based on the model using the inferred parameters of the EM, the rEM, the rEM_*w*_, and the true gene expression is shown in Figure 25. The left top and right top panels are the expression of the first gene and the second gene, respectively. The left center and right center panels are the expression of the third gene and the fourth gene, respectively. The left bottom and right bottom panels are the expression of the fifth gene and the sixth gene, respectively. It can be seen that the estimate gene expression using the UKF and parameters given by the EM, the rEM, and the rEM_*w*_ is close to the true gene expression data. In addition, The rEM_*w*_ algorithm provide sparser solution than the rEM algorithm. Both the rEM and the rEM_*w*_ algorithms give sparser solutions than the EM algorithm which validates the effectiveness of the proposed method.

## 6 Conclusions

In this paper, we have considered the problem of sparse parameter estimation in a general nonlinear dynamic system, and we have proposed an approximate MAP-EM solution, called the rEM algorithm. The expectation step involves the forward Gaussian approximation filtering and the backward Gaussian approximation smoothing. The maximization step employs a re-weighted iterative thresholding method. We have provided examples of the inference of gene regulatory network based on expression data. Comparisons with the traditional EM algorithm as well as with the existing approach to solving sparse problems such as the *ℓ*_1_ optimization and the BPDN-DF show that the proposed rEM algorithm provides both more accurate estimation result and sparser solutions.

## Competing interests

The authors declare that they have no competing interests.
